# Knowledgeable, aware / interested: Young black women's perceptions of pre-exposure prophylaxis

**DOI:** 10.3389/frph.2022.671009

**Published:** 2022-09-30

**Authors:** Sadia Haider, Eleanor E. Friedman, Emily Ott, Amy Moore, Agustina Pandiani, Catherine Desmarais, Amy K. Johnson

**Affiliations:** ^1^ Department of Obstetrics and Gynecology, Rush University Medical Center, Chicago, IL, United States; ^2^ Department of Obstetrics and Gynecology, University of Chicago, Chicago, IL, United States; ^3^ Division of Adolescent Medicine, Ann / Robert H. Lurie Children’s Hospital of Chicago, Chicago, IL, United States; ^4^ Department of Pediatrics, Northwestern Feinberg School of Medicine, Chicago, IL, United States

**Keywords:** women’s health services, hiv prevention, women, adolescent, black, pre-exposure prophylaxis

## Abstract

**Purpose:**

HIV in the United States disproportionately affects young Black women. Pre-exposure prophylaxis (PrEP) is an effective HIV prevention option that has the potential to reduce HIV incidence among HIV-vulnerable populations. However, data regarding women's awareness, interest in starting, and feelings of acceptability or stigma about PrEP remains limited, particularly among adolescent and young Black women.

**Materials and methods:**

A cross-sectional survey was conducted with 100 sexually active young Black women ages 13–24 years attending women's health clinics in Chicago, IL. Descriptive statistics were used to characterize the sample and determine more about what the PrEP needs and barriers are in this community. Associations were modeled using logistic regression and 95% confidence intervals for both bivariate and multivariable models.

**Results:**

In our survey (*N* = 100), half of study participants (50%) expressed interest in starting PrEP in the next three months and a majority (80%) of young women were confident they could obtain PrEP. Pregnant young women were significantly more interested in starting PrEP than non-pregnant women [OR 2.3 95% CI (1.0, 5.4)], *p* = 0.05), however, this association did not remain significant in adjusted models.

**Conclusions:**

This study provides a more complete understanding of awareness, interest in, and acceptability of PrEP among adolescent and young Black women attending women's health clinics. Findings indicate sustained interest in starting PrEP, reduced stigma, and increased awareness of PrEP among young Black women. These findings suggest that integrating PrEP into women's health clinics is a promising strategy to increase awareness and utilization of PrEP and decrease HIV transmission among youth at highest risk.

## Introduction

Young Black women are disproportionately burdened by HIV infection in the United States, accounting for the highest rate of HIV diagnoses among young people aged 13–29 years (1). Specific to Chicago, youth ages 13–29 represented 43.7% of all new HIV infections in 2018, more than any other age group (2). In fact, young Black women acquire HIV at rates 15 times that of young White women (3, 4).

First approved in 2012 for adult populations and then in May 2018 for high-risk adolescents, pre-exposure prophylaxis (PrEP) is an effective prevention strategy that can reduce HIV infection among HIV-negative populations (5–7). Additionally, unlike other prevention strategies like condoms, PrEP allows women to have greater agency over their sexual lives since it does not require a sexual partner's permission or cooperation for use (8–10). Despite PrEP's demonstrated effectiveness, women remain under-represented in HIV prevention efforts using this method (9), as much of the work related to PrEP has focused on men who have sex with men (11). Furthermore, data regarding awareness and provision of PrEP among adolescent and young women remains limited (12).

In 2015, the Centers for Disease Control and Prevention estimated that 468,000 women in the United States were at risk of being infected with HIV *via* heterosexual contact (13). However, less than 5% of PrEP users in the United States in 2016 were women, accounting for just 2% of the estimated 176,670 heterosexual women indicated to be PrEP-eligible (13–15). Furthermore, only 11% of PrEP prescriptions were for patients under the age of 25 and only 4.6% were among women (15). Given this underuse of PrEP by young women, it is critical to understand what factors, including, system-, provider- and patient-level factors, may be influencing both PrEP awareness and uptake for this population.

Specifically, additional work is needed among young Black women to better understand their interest and needs in relation to PrEP. Research to date highlights the multi-level factors influencing awareness and uptake of PrEP (16). In a recent qualitative study of young adult Black women in New York City, focus group participants identified cultural-environmental factors (e.g., a lack of information and resources to access PrEP), socio-normative factors (e.g., attitudes towards the long-term effects of PrEP and self-efficacy to follow the regimen), and proximal interpersonal factors (e.g., perceived HIV stigma from family and peers and a fear of rejection from partners) affecting PrEP awareness and uptake (17). In a study of 1,621 Black women age 14–24 years recruited from community venues and sexual health clinics in Atlanta, findings reiterate both the high risk level among this population and the challenge of identifying women at risk of HIV (18). In this sample, many women did not report any behavioral risk factors, underpinning the need to adapt current HIV risk assessments for women. PrEP acceptability was moderate; 43% of the community sample reported that they would be very likely to use PrEP. This is supported in studies of adolescents, with high acceptability scores reported among adolescents age 13–17 (19). Shah and colleagues (2019) found no significant differences in acceptability by sexual activity or age (16 and older vs. younger than 16) (19). Among sexually active adolescents, adolescents who did not use condoms during last sexual intercourse or who used them intermittently were more likely to find PrEP acceptable (19). Adolescents in this case reported moderate barriers to PrEP. Among sexually active adolescents, non-Black adolescents had significantly fewer perceived barriers to PrEP.

Previous work with HIV-vulnerable women indicates that women's health clinics provide key access points to reach women at higher risk for HIV infection (20). Women's health clinics are an important source of sexual and reproductive health for adolescents and young adults. Visits to women's health clinics provide an opportunity to educate adolescents on sexual health and development and discuss prevention of sexually transmitted infections (STIs) and HIV (21). Women's health providers are often one of the only touchpoints within the healthcare system for women, with 40% of women in the United States exclusively accessing reproductive healthcare (22). Among young women the majority reported accessing sexual and reproductive health clinics for contraceptive or STI services at least once in the last year (23, 24). As such, women's health providers are in a unique position to address women's HIV risk perception and ensure women are aware of and offered comprehensive HIV prevention options (20), especially for young Black women who are at higher risk of infection (21).

Both the Centers for Disease Control and Prevention and the Office of Population Affairs identify HIV prevention, including PrEP, as a core family planning service (25). Black women have expressed greater comfort and willingness to discuss PrEP with their healthcare providers than White women, and women with higher HIV risk such as those with multiple partners were more amenable to PrEP use (26). Therefore, integration of HIV prevention and PrEP education into regular women's healthcare services for all patients, and particularly young women, is a natural extension of sexual health care.

Women's awareness, attitudes and opinions of PrEP have not been studied extensively in the United States (27). In addition, little research to date has been conducted among young Black women to better understand their interest and needs in relation to PrEP. Engaging adolescent and young Black women in the development of tailored approaches to PrEP education and provision efforts is critical to reducing HIV-related health disparities. This study aimed to (1) assess awareness of PrEP among young Black women and (2) examine barriers and facilitators to PrEP uptake among adolescent and young Black women accessing women's health clinics in Chicago.

## Materials and methods

### Data collection

An observational cross-sectional survey was conducted with young Black women attending women's health clinics at either the University of Chicago Ryan Center or at a Planned Parenthood Illinois Health Center. Participants were eligible if they were: English speaking, self-identified as African American or Black, cisgender female, between ages 13–24 years, live in Chicago, and reported sexual activity within the previous six months. In addition, participants if they were currently taking PrEP and if they answered “Yes” were ineligible to participate in the survey. All participants over 18 years completed oral informed consent prior to engaging in the study. Oral informed assent was obtained for participants under the age of 18 years and a parental waiver of consent for minor participants was granted to protect privacy of participants. Following survey completion, participants received $50 compensation and a list of local PrEP-related resources. Survey data were collected and managed using REDCap electronic data capture tools hosted at University of Chicago (28, 29). The Institutional Review Boards from the University of Chicago (IRB18-0901) and Lurie Children's Hospital (IRB 2019-2586) reviewed and approved the study.

Survey procedures were completed in a private room or private area of the clinic waiting room and consisted of a quantitative survey and a series of semi-structured open-ended questions. Surveys were administered by research assistants and all responses were entered into REDCap using a handheld device. All data were self-reported. The survey contained 91 items, including 4 items in an open-ended format, and was designed to capture information about sexual health and behavior, PrEP awareness, acceptability, barriers and facilitators to uptake, as well as demographic and behavioral domains. When available, we used published survey items from the existing literature, including items on PrEP knowledge, attitudes, and PrEP stigma (30–32); when not available we modified existing measures and survey items to fit our study population (in terms of age and sex). Demographic characteristics included age, sex at birth, gender identity, race/ethnicity, currently pregnant, history of abortions in the last 12 months, HIV status, medical insurance information, neighborhood of residence, education history, and type of medical appointment. Age was categorized into the following groups; ≤18, 19–22, and >22 years of age based on clinical expertise and informed by national guidance (33). Sexual health was measured with questions such as: “In the last 3 months, have you been treated for or received medication from a doctor or medical care provider for an STI?”. Sexual behavior was measured by a series of items assessing whether a behavior occurred (e.g., “Have you had vaginal and/or anal sex in the past 6 months?”). Condom use was assessed by asking “In the last 3 months, how often did you or your partner(s) use condoms (Always, sometimes/never, I have not had sex in the last three months). Self-perceived HIV risk was assessed with two items, specifically “I think my chances of getting HIV are”; with response options of 0%, no chance I will get HIV, 25%, 50%, 75%, and 100%, I definitely will get HIV; and “How likely are you to become infected with HIV, or infect others, based on your current sexual practices?” with 5 Likert choices from very unlikely to very likely. Assessment of HIV as a general threat to the African American community was measured by the statement “I am concerned about high rates of HIV in the African American/Black community:” with response options of agree, undecided and disagree. Personal attitudes towards PrEP use were measured by statements such as “I would get frustrated taking PrEP because I would have to plan my life around it”, “I don't like the thought of taking PrEP because it reminds me of HIV”, “It frustrates me to think that I would have to take PrEP every day in order for it to be effective”, and “I am worried taking PrEP will hurt my health” with five category Likert response scales from strongly disagree to strongly agree. PrEP stigma was measured by statements like “PrEP will cause people to have more risky sex”, “Instead of taking PrEP, people should just pick their partners carefully”, and “PrEP is for people who are promiscuous (e.g., “slutty” or “easy”)” with the same five category Likert response detailed above. PrEP access and comfort was assessed with the questions “If you were interested in taking PrEP, how confident would you be in your ability to obtain it?”, “Do you feel like PrEP is something you could talk about with a partner (current or future)?”, and “Do you feel like PrEP is something you could talk about with a parent/guardian(s)?” with options of Definitely or probably could, definitely or probably could not, and I’m not sure if I could or could not. PrEP awareness was assessed via a single item, “Prior to this study, had you heard of PrEP or the use of medication to prevent HIV infection?”. Source of PrEP knowledge was examined by asking “Where did you hear of PrEP before this study?” and allowing participants to select among a list of possible sources of PrEP knowledge. PrEP acceptability was assessed via a single item, specifically, “Knowing PrEP is highly effective in preventing HIV, how likely would you be to take it?” with high prep acceptability being very likely/likely to take PrEP. Interest in starting PrEP was assessed by the question “How likely are you to start taking PrEP in the next 3 months?” with answers dichotomized into the categories of very likely/likely or unlikely/somewhat unlikely. We examined perceived barriers to PrEP with “Which of the following are barriers for you to PrEP uptake?” with the ability to select multiple possible barriers. Outcomes of interest included having heard of PrEP prior to this study and interest in starting PrEP.

### Analysis

Descriptive statistics were used to characterize the sample and bivariate analysis was used to detect differences between categorical outcome variables using chi-square tests. Bivariate and multivariate logistic regression models were created, resulting in odds ratios (OR) or adjusted odds ratios (aOR) and 95% confidence intervals for the outcomes of awareness of PrEP and interest in starting PrEP. As our study population contained both adolescents and young adults, we decided to examine all variables after stratifying or adjusting for age group. Therefore, adjusted logistic regression models include all variables found to be statistically significant at the bivariate level (*p* = 0.05) as well as age group. Quantitative analysis was conducted in SAS 9.4 (SAS Institute Inc., Cary, NC, USA).

## Results

A total of 100 young women completed the study from January 2019 through June 2019. Participants had a median age of 22 years (interquartile interval 20–23) with 13% less than or equal to 18 years of age, 37% between 19 and 22 years of age, and 50% over 22 years of age. Women were at the clinic to accompany a friend or family member (15%), for a women's healthcare visit (32%), STI testing or treatment (5%), an abortion (12%), a prenatal visit (10%), or did not disclose (26%) (Table 1). Over a third of the sample was currently pregnant (37%), while 20% had undergone one or more abortions in the last year. Nearly all women were sexually active in the past three months (vaginal sex 99%, anal sex 18%) often using condoms inconsistently (inconsistent condom use for vaginal sex 92%, and for anal sex 94%). Young women had low perceived risk of HIV infection, with 88% rating their chances of getting HIV as 25% or less, with only 8% of respondents saying they were likely to become infected with HIV or infect others, based on their current sexual practices.

**Table 1:** Demographics and survey responses for study participants, separated by age group.

Variable	**≥18** Number (percentage) (*N* = 13)	**19-22** Number (percentage) (*N* = 37)	**Over 22** Number (percentage) (*N* = 50)	All ages Number (percentage) (*N* = 100)
Hispanic				
Yes	0 (0.0%)	0 (0.0%)	1 (2.0%)	2 (2.0%)
No	13 (100.0%)	35 (94.6%)	49 (98.0%)	97 (97.0%)
Missing	0 (0.0%)	1 (5.4%)	0 (0.0%)	1 (1.0%)
African American				
Yes	13 (100.0%)	35 (94.6%)	50 (100.0%)	98 (98.0%)
Biracial	0 (0.0%)	2 (5.4%)	0 (0.0%)	2 (2.0%)
Gender Identity				
Identify as female	13 (100.0%)	37 (100.0%)	49 (98.0%)	99 (99.0%)
Identify as genderqueer or non-conforming	0 (0.0%)	0 (0.0%)	1 (2.0%)	1 (1.0%)
Pregnant				
Yes	5 (38.5%)	14 (37.8%)	18 (36.0%)	37 (37.0%)
No	8 (61.5%)	23 (62.2%)	27 (54.0%)	58 (58.0%)
Don’t know	0 (0.0%)	0 (0.0%)	5 (10.0%)	5 (5.0%)
Any abortions in the past 12 months				
No	12 (92.3%)	29 (78.4%)	39 (78.0%)	80 (80.0%)
Yes	1 (7.7%)	8 (21.6%)	11 (22.0%)	20 (20.0%)
Living with HIV				
No	13 (100.0%)	36 (97.3%)	49 (96.0%)	97 (97.0%)
Don’t know	0 (0.0%)	1 (2.7%)	2 (4.0%)	3 (3.0%)
Medical insurance				
Medicaid/Uninsured	10 (76.9%)	27 (73.0%)	30 (60.0%)	67 (67.0%)
Private insurance/employer sponsored	3 (23.1%)	6 (16.2%)	19 (38.0%)	28 (28.0%)
Missing	0 (0.0%)	4 (10.8%)	1 (2.0%)	5 (5.0%)
Neighborhood				
Northside	2 (15.4%)	5 (13.5%)	4 (8.0%)	11 (11.0%)
Southside	9 (69.2%)	19 (51.4%)	35 (70.0%)	63 (63.0%)
Westside	1 (7.7%)	4 (10.8%)	3 (6.0%)	8 (8.0%)
Outside of Chicago	0 (0.0%)	8 (21.6%)	7 (14.0%)	15 (15.0%)
Missing	1 (7.7%)	1 (2.7%)	1 (2.0%)	3 (3.0%)
Number of years of education				
High school/GED or fewer	12 (92.3%)	17 (46.0%)	18 (36.0%)	47 (47.0%)
Above high school or GED	1 (7.7%)	20 (54.1%)	32 (64.0%)	53 (53.0%)
How often did you or your partner (s) use condoms during anal sex?^a,b^				
Always	1 (33.3%)	0 (0.00%)	0 (0.0%)	1 (5.6%)
Sometimes or never	2 (66.7%)	5 (100.0%)	10 (100.0%)	17 (94.0%)
I have not had anal sex^a^	10	32	40	82
How often did you or your partner (s) use condoms during vaginal sex?^a,c^				
Always	1 (7.7%)	3 (8.1%)	3 (6.1%)	7 (7.1%)
Sometimes or never	12 (92.3%	34 (91.9%	46 (93.9%	92 (92.9%
I have not had vaginal sex^a^	0	0	1	1
Awareness of PrEP				
Prior to this study, have you heard of PrEP (Pre-exposure prophylaxis) or the use of medication to prevent HIV infection?				
Yes	6 (46.2%)	22 (59.5%)	25 (50.0%)	53 (53.0%)
No	7 (53.9%)	15 (40.5%)	25 (50.0%)	47 (47.0%)
Interest in starting PrEP				
How likely are you to start taking PrEP in the next 3 months?				
Very likely or likely	7 (53.9%)	17 (46.0%)	26 (52.0%)	50 (50%)
Unlikely or somewhat unlikely	6 (46.2%)	20 (54.1%)	24 (48.0%)	50 (50%)
Acceptability of PrEP				
Knowing PrEP is highly effective in preventing HIV, how likely would you be to take it?				
Very likely or likely	13 (100.0%)	32 (86.5%)	43 (86.0%)	88 (88.0%)
Unlikely or somewhat unlikely	0 (0.0%)	5 (13.5%)	7 (14.0%)	12 (12.0%)
I would get frustrated taking PrEP because I would have to plan my life around it.				
Somewhat disagree or strongly disagree	6 (46.2%)	28 (75.7%)	36 (72.0%)	70 (70.0%)
Somewhat agree or strongly agree	7 (53.9%)	6 (16.2%)	10 (20.0%)	23 (23.0%)
Neither agree nor disagree	0 (0.00%)	3 (8.1%)	4 (8.0%)	7 (7.0%)
I don’t like the thought of taking PrEP because it reminds me of HIV.				
Somewhat disagree or strongly disagre	6 (46.2%)	30 (81.1%)	38 (76.0%)	74 (74.0%)
Somewhat agree or strongly agree	4 (30.8%)	6 (16.2%)	10 (20.0%)	20 (20.0%)
Neither agree nor disagree	3 (23.1%)	1 (2.7%)	2 (4.0%)	6 (6.0%)
It frustrates me to think that I would have to take PrEP every day in order for it to be effective.				
Somewhat disagree or strongly disagree	7 (53.9%)	26 (70.3%)	27 (54.0%)	60 (60.0%)
Somewhat agree or strongly agree	4 (30.8%)	7 (18.9%)	17 (34.0%)	28 (28.0%)
Neither agree nor disagree	2 (15.4%)	4 (10.8%)	6 (12.0%)	12 (12.0%)
I am worried taking PrEP will hurt my health.				
Somewhat disagree or strongly disagree	5 (38.5%)	17 (46.0%)	27 (54.0%)	49 (49.0%)
Somewhat agree or strongly agree	6 (46.2%)	11 (29.7%)	12 (24.0%)	29 (29.0%)
Neither agree nor disagree	2 (15.4%)	9 (24.3%)	11 (22.0%)	22 (22.0%)
PrEP will cause people to have more risky sex.				
Somewhat disagree or strongly disagree	2 (15.4%)	10 (27.0%)	20 (40.0%)	32 (32.0%)
Somewhat agree or strongly agree	9 (69.2%)	16 (43.2%)	25 (50.0%)	50 (50.0%)
Neither agree nor disagree	2 (15.4%)	11 (29.7%)	5 (10.0%)	18 (18.0%)
Instead of taking PrEP, people should just pick their partners carefully.				
Somewhat disagree or strongly disagree	4 (30.8%)	12 (32.4%)	19 (38.0%)	35 (35.0%)
Somewhat agree or strongly agree	7 (53.9%)	14 (37.8%)	13 (26.0%)	34 (34.0%)
Neither agree nor disagree	2 (15.4%)	11 (29.7%)	18 (36.0%)	31 (31.0%)
PrEP is for people who are promiscuous (e.g., “slutty” or “easy”)				
Somewhat disagree or strongly disagree	6 (46.2%)	29 (78.4%)	46 (92.0%)	81 (81.0%)
Somewhat agree or strongly agree	4 (30.7%)	2 (5.4%)	2 (4.0%)	8 (8.0%)
Neither agree nor disagree	3 (23.1%)	6 (16.2%)	2 (4.0%)	11 (11.0%)
If you were interested in taking PrEP, how confident would you be in your ability to obtain it?				
Definitely or probably could	9 (69.2%)	28 (75.7%)	43 (86.0%)	80 (80.0%)
Definitely or probably could not	1 (7.7%)	2 (5.4%)	3 (6.0%)	6 (6.0%)
I’m not sure if I could or could not	3 (23.1%)	7 (18.9%)	4 (8.0%)	14 (14.0%)
Do you feel like PrEP is something you could talk about with a partner (current or future)?				
Definitely or probably could	10 (76.9%)	33 (89.2%)	47 (94.0%)	90 (90.0%)
Definitely or probably could not	1 (7.7%)	2 (5.4%)	2 (4.0%)	5 (5.0%)
I’m not sure if I could or could not	2 (15.4%)	2 (5.4%)	1 (2.0%)	5 (5.0%)
Do you feel like PrEP is something you could talk about with a parent/guardian (s)?				
Definitely or probably could	10 (76.9%)	30 (81.1%)	38 (76.0%)	78 (78.0%)
Definitely or probably could not	1 (7.7%)	6 (16.2%)	10 (20.0%)	17 (17.0%)
I’m not sure if I could or could not	2 (2.0%)	1 (2.7%)	2 (4.0%)	5 (5.0%)
How likely are you to become infected with HIV, or infect others, based on your current sexual practices?				
Very unlikely or unlikely	12 (92.3%)	35 (94.6%)	45 (90.0%)	92 (92.0%)
Somewhat likely or likely	1 (7.7%)	2 (5.4%)	5 (10.0%)	8 (8.0%)
I think my chances of getting HIV are:				
50%–100%	0 (0.0%)	5 (13.5%)	7 (14.0%)	12 (12.0%)
Less than 50%	13 (100.0%)	32 (86.5%)	43 (86.0%)	88 (88.0%)
I am concerned about high rates of HIV in the African American/Black community:				
Agree	10 (76.9%)	26 (70.3%)	44 (88.0%)	80 (80.0%)
Undecided	1 (7.7%)	7 (18.9%)	2 (4.0%)	10 (10.0%)
Disagree	2 (15.4%)	4 (10.8%)	4 (8.0%)	10 (10.0%)
Which of the following are barriers for you to PrEP uptake? (select all)				
Lack of communication among community members	1 (7.7%)	4 (10.8%)	1 (2.0%)	6 (6.0%)
Mistrust of the medical community	0 (0.0%)	4 (10.8%)	3 (6.0%)	7 (7.0%)
Cost	1 (7.7%)	8 (21.6%)	12 (24.0%)	21 (21.0%)
Side effects	5 (38.5%)	20 (54.1%)	21 (42.0%)	46 (46.0%)
Stigma	0 (0.0%)	0 (0.0%)	2 (4.0%)	2 (2.0%)
Drug is too new	2 (15.4%)	4 (10.8%)	2 (4.0%)	8 (8.0%)
Lack of housing	0 (0.0%)	0 (0.0%)	0 (0.0%)	0 (0.0%)
Fear of using parental insurance	0 (0.0%)	1 (2.7%)	3 (6.0%)	4 (4.0%)
Where did you hear of PrEP before this study?^d^ (select all)				
TV/Radio	1 (14.3%)	7 (36.8%)	13 (48.1%)	21 (39.6%)
Health Care Professional	3 (42.9%)	6 (31.6%)	8 (29.6%)	17 (32.1%)
Friends/Family/Acquaintance	2 (28.6%)	5 (26.3%)	7 (25.9%)	14 (26.4%)
Social media platforms	1 (14.3%)	3 (15.8%)	8 (29.6%)	12 (22.6%)
Classes at school/ sex education classes	2 (28.6%)	4 (21.1%)	4 (14.8%)	10 (18.9%)
Chicago Transit Authority ads	0 (0.0%)	1 (5.3%)	6 (22.2%)	7 (13.2%)
On a website	0 (0.0%)	2 (10.5%)	2 (7.4%)	4 (7.5%)
Newspapers/Magazines	0 (0.0%)	2 (10.5%)	1 (3.7%)	3 (5.7%)
At a community meeting	0 (0.0%)	1 (5.3%)	0 (0.0%)	1 (1.9%)
A sex partner	0 (0.0%)	0 (0.0%)	0 (0.0%)	0 (0.0%)
HIV Service Agency	0 (0.0%)	0 (0.0%)	0 (0.0%)	0 (0.0%)
Had not heard of PrEP before today	6	18	23	47

^a^ In the last 3 months.

^b^ Percentages are out of those who had anal sex.

^c^ Percentages are out of those who had vaginal sex.

^d^ Percentages are out of those who had heard of PrEP.

Over half of participants (53%) were aware of PrEP prior to taking this survey. Most common sources of having heard of PrEP included from the TV/Radio (40%), from a healthcare professional (32%), from a friend/acquaintance or family member (26%), from social media platforms (26%), or from high-school, college or job training (19%) classes (Table 1).

About a third of young women in this study expressed concerns regarding the mechanics of taking PrEP, including needing to take it daily (28.0%), being reminded of HIV when taking PrEP (20.0%) and concerns regarding potential health risks of PrEP (29.0%). The largest perceived barrier to PrEP uptake were side effects (46.0%), followed by cost (21.0%). Young women in our study largely rejected the idea that taking PrEP meant you were promiscuous (81.0%), but about half felt that using PrEP would result in risk compensation (50.0%) and about a third suggested that instead of taking PrEP, people should just choose their partners more carefully (34.0%). In particular, participants less than or equal to 18 years of age were more concerned that PrEP might promote promiscuity (31%) or result in risk compensation (69%), and were more inclined to think that instead of PrEP use, people should choose different partners (53%). The youngest participants also expressed more concerns related to the requirement to plan for and the daily nature of PrEP as an HIV preventative (“It frustrates me to think that I would have to take PrEP every day in order for it to be effective” (31%); “I would get frustrated taking PrEP because I would have to plan my life around it.” (54%).

Overall, young women seemed comfortable with PrEP and did not evidence stigma regarding PrEP usage. Young women expressed willingness to discuss PrEP with parents (78%) and partners (90%). Only 4.0% of women expressed that using parental insurance would be a barrier to them obtaining PrEP, and only 2% listed stigma as a barrier. Half of study participants expressed a willingness to start PrEP in the next three months, and the majority of young women were confident they could obtain PrEP (80%) (Table 1).

When associations between interest in starting PrEP and perceptions, awareness, attitudes and acceptability of PrEP were examined, participants who reported high PrEP acceptability had 13 times the odds of interest in starting PrEP [OR 13.8 95% CI (1.7, 111.7)]. Participants being aware of PrEP prior to this study had 0.3 times the odds of being interested in starting PrEP [OR 0.3 95% CI (0.2, 0.8)], and participants who felt PrEP reminded them of HIV had 0.3 times the odds of interest in starting PrEP [OR 0.3 95% CI (0.1, 0.9)]. Participants who were unsure if taking PrEP would remind them of HIV did not display a significant association [OR 0.4 95% CI (0.1, 2.2)]. These associations persisted in strength and direction in a multivariate model that adjusted for age groups. In adjusted models high PrEP acceptability was still associated with 13 times the odds of interest in starting PrEP [aOR 13.7 95% CI (1.4, 130.9)], and participants aware of PrEP prior to this study had 0.4 times the odds of starting PrEP [aOR 0.4 95% CI (0.1, 0.9)] (Table 2). Pregnant participants initially had 2.3 times the odds of interest in starting PrEP [OR 2.3 95% CI (1.0, 5.4)], however this association did not remain significant after adjusting for other factors (Table 2).

**Table 2:** Statistically significant associations between study variables and awareness of PrEP or interest in starting PrEP adjusted for age group.

	OR (95% CI)	Predicted probabilities	*p*-value	aOR (95% CI)	Predicted probabilities	*p*-value
Awareness of PrEP						
Age categories*						
≥18	Referent	0.5	Referent	Referent	0.4	Referent
19–22	1.7 (0.5, 6.1)	0.6	0.4	1.8 (0.5, 6.6)	0.6	0.4
Over 22	1.2 (0.3, 4.0)	0.5	0.8	1.4 (0.1, 5.0)	0.5	0.6
Pregnancy						
Not pregnant	Referent	0.7	Referent	Referent	0.4	Referent
Pregnant	0.3 (0.1, 0.8)	0.4	0.01	3.1 (1.3, 7.4)	0.6	0.01
Interest in starting PrEP						
Age categories*						
>18	Referent	0.5	Referent	Referent	0.2	Referent
19–22	0.7 (0.2, 2.6)	0.5	0.6	0.5 (0.1, 2.0)	0.2	0.3
Over 22	0.9 (0.3, 3.2)	0.5	0.9	0.6 (0.1, 2.5)	0.2	0.8
Pregnancy						
Not pregnant	Referent	0.4	Referent	Referent	0.2	Referent
Pregnant	2.3 (1.0, 5.4)	0.6	0.05	1.9 (0.8, 4.9)	0.1	0.2
PrEP acceptability						
Unlikely or somewhat unlikely	Referent	0.1	Referent	Referent	0.2	Referent
Very likely or likely	13.8 (1.7, 111.7)	0.6	0.01	13.7 (1.4, 130.9)	0.4	0.03
Awareness of PrEP						
No	Referent	0.6	Referent	Referent	0.2	Referent
Yes	0.3 (0.2, 0.8)	0.4	0.01	0.4 (0.1, 0.9)	0.1	0.04
I don’t like the thought of taking PrEP because it reminds me of HIV						
Disagree	Referent	0.6	Referent	Referent	0.2	Referent
Agree	0.3 (0.1, 0.9)	0.3	0.04	0.3 (0.1, 0.9)	0.2	0.5
Unsure	0.4 (0.1, 2.2)	0.3	0.3	0.2 (0.1, 1.4)	0.1	0.3

* Entered into the models *a priori*, without regard to statistical significance.

When associations were examined between PrEP awareness prior to the study and demographic or behavioral factors, only currently being pregnant was found to be significant. Pregnant women had 0.32 times the odds of being aware of PrEP prior to this study [OR 0.3 95% CI (0.1, 0.8)]. However, in a model adjusting for age group, the association between pregnancy and awareness of PrEP changed direction with pregnant women having 3 times the odds of being aware of PrEP prior to this study [aOR 3.1 95% CI (1.3, 7.4)]. (Table 2).

## Discussion

This is one of the first studies to explore PrEP awareness and interest among adolescent and young Black women attending women's health clinics in urban areas with high HIV prevalence. This study found relatively high levels of PrEP awareness, acceptance, and interest in starting PrEP among young Black women with half of study participants expressing interest in starting PrEP in the next three months and a majority (80%) of young women reporting that they were confident they could obtain PrEP. A strength of this study was that our population of young Black women reflected a high risk female demographic and our results build on a previously conducted study by the same investigative team among adult Black women attending women's health clinics. Our previous study found 35% of adult Black women had heard of PrEP (34) and in comparison, over half (53%) of young Black women in this study were aware of PrEP. This may be in part due to the young age of participants and generational differences in information sources given PrEP is a recent innovation for HIV prevention since 2012 (35). For instance, among young Black participants in this study, television, social media, and education received from classes in high school, college or job training were commonly listed as sources that provided PrEP information.

In addition, PrEP acceptability or interest in starting PrEP has been shown to be significantly higher among Black women than among White women, with 71% of Black women at a family planning clinic in Baltimore indicating they would take PrEP to prevent HIV infection (21) and both Black mothers and their daughters in Chicago reported wishing they had been made aware of PrEP (36). Our own findings also reflect high acceptability, with 88% of young Black women reporting PrEP acceptability, and 50% being interested in starting PrEP within the next three months. We also found that pregnant women were significantly more interested in starting PrEP than non-pregnant women. This may be a result of increased self-awareness as a result of pregnancy and increased interactions with the health care setting.

Previous studies have demonstrated women have low awareness of their risk for HIV acquisition (37, 38). In our study, 88% of participants do not consider themselves at risk for HIV despite also reporting they are concerned about high rates of HIV in the African American/Black community (80%). Given the high rates of unprotected sex and history of STIs reported in our study, efforts should be made to inform and counsel women about HIV acquisition risk factors with a focus on heterosexual transmission, since 85% of new HIV diagnoses among women were attributed to heterosexual contact in 2018 (4, 39). Since many young women seek sexual and reproductive health care, women's health care providers can play a pivotal role in identifying women who would benefit from PrEP and linking them to HIV prevention services. Furthermore, the main barriers to PrEP uptake in this study were side effects (46%) and cost (21%) which are commonly reported barriers in PrEP-related studies conducted among adult Black women (8, 37, 38, 40). Women's health clinics are an ideal setting to provide anticipatory counseling regarding side effects and other health concerns related to PrEP initiation. In addition, these settings can provide PrEP education materials and navigation assistance (e.g., payment assistance programs, referrals, etc).

Low perception of stigma was reported by study participants, a finding quite different from previous research with adult women (40, 41). Possible reasons for this include the young age of participants and the high percentage of participants who reported hearing of PrEP from sources of authority such as health care providers or as part of an educational or vocational class. Similarly, the high percentage of participants who reported learning of PrEP from friends and family or social media could also be responsible for reduced PrEP stigma in this population. A lack of worry about judgment and comfort with PrEP is also illustrated by the fact that 78% of young women were comfortable discussing PrEP with their parents and 90% were comfortable discussing it with partners. Similarly, a study conducted with parents and their adolescent daughters, discovered 92% of parents of Black urban adolescent girls attending a pediatric and adolescent clinic knew they were using contraceptives, and 87% of adolescent girls had engaged in one or more conversations with their parents about sex (42). In addition, a study with Black mothers found that they were aware that their daughters will engage in sexual activity at some point and are supportive of PrEP use as a preventative measure (36). It is very promising to see parental and partner disclosure of PrEP being reported favorably among adolescent and young women since stigma and disclosure concerns have been key barriers to PrEP uptake and adherence for women in the past (43).

Findings should be interpreted in light of study limitations. First, a small sample size limited statistical power, particularly for results of our multivariable logistic models. This may have led to the inability to detect differences in subgroups for variables with smaller effect sizes. Additionally, our population was somewhat homogenous, as women were recruited from only two women's health clinics and exclusively among participants of one racial group and of a limited age range. We also did not collect information on income or qualification for federal poverty level, limiting our ability to analyze concerns related to PrEP cost. These sample limitations reduce generalizability of findings to all women's health patients. Finally, all data were self-reported and may be subject to social desirability bias.

## Conclusions

This study provides a more complete understanding of awareness and acceptability of PrEP among adolescent and young Black women attending women's health clinics. Our findings suggest that adolescent and young Black women demonstrate sustained interest in starting PrEP and reduced stigma related to PrEP uptake. In addition, younger Black women are more likely to have become aware of PrEP *via* social media and network dissemination and this higher awareness could be leveraged during their visit with a women's health professional. Integrating PrEP education and provision services in women's health clinics can serve as a promising strategy to improve uptake of PrEP among adolescent and young Black women and to address low rates perceived vulnerability of HIV acquisition. Further research is needed to develop youth-friendly approaches to improve PrEP scale-up initiatives for populations at highest risk.

## Data Availability

The raw data supporting the conclusions of this article will be made available by the authors, without undue reservation.
